# Low PAPP-A levels and their association with adverse perinatal outcomes in twin pregnancies

**DOI:** 10.1007/s00404-025-08299-7

**Published:** 2026-01-19

**Authors:** Sapantzoglou Ioakeim, Afroditi Maria Kontopoulou, Karasmani Christina, Fasoulakis Zacharias, Maria Ioanna Chatziioannou, Pegkou Afroditi, Simou Maria, Pafilis Ioannis, Souka Athina, Theodora Marianna, Antsaklis Panagiotis, Daskalakis Georgios

**Affiliations:** 1 Department of Obstetrics and Gynecology, General Hospital Alexandra, National and Kapodistrian University of Athens, Lourou 4-2, 15238 Athens, Greece; 2 Unit of Fetoscopy, Fetal Therapy and Laser Applications, Department of Obstetrics and Gynecology, General Hospital Alexandra, National and Kapodistrian University of Athens, 15238 Athens, Greece; 3 Iatriki Embryou-Ioannis Pafilis, 10676 Athens, Greece

**Keywords:** PAPP-A, Twin pregnancy, Adverse pregnancy outcomes, PTB, PET

## Abstract

**Purpose:**

While in singleton pregnancies the maternal serum biomarkers used in the first-trimester screening for aneuploidies, particularly PAPP-A, may be, also, used as predictors of adverse perinatal outcomes, there is a scarcity of data regarding the association of first-trimester biomarkers with unfavorable pregnancy outcomes in twin pregnancies. The main purpose of our study was to evaluate the association of low PAPP-A levels in twin pregnancies with the subsequent development of perinatal complications.

**Methods:**

454 twin pregnancies were recruited over a period of 11 years, and their data were analyzed retrospectively. First trimester assessment at 11 + 0–13 + 6 weeks included examination of fetal anatomy and markers of aneuploidy and measurement of the maternal serum concentration of PAPP-A and free b-hCG. The outcomes under investigation were preterm rupture of the membranes, preterm delivery earlier than 32, 34, and 36 weeks, gestational diabetes, hypertensive disease of the pregnancy (including pregnancy induced hypertension and pre-eclampsia), intrauterine demise, birth weight difference of more than 25% among the fetuses and composite adverse pregnancy outcome (which included preterm rupture of the membranes, preterm delivery earlier than < 36 weeks, hypertensive disease of the pregnancy, and gestational diabetes).

**Results:**

Low first-trimester PAPP-A levels were related with preterm birth, whereas high levels were associated with hypertensive disorders of pregnancy. When using specific cut-off points such as low PAPP-A MoM < 5th and < 10th centile or high PAPP-A MoM > 90th and 95th centile, these associations were not significant.

**Conclusions:**

Low first-trimester PAPP-A levels were related with preterm birth, whereas high levels were associated with hypertensive disorders of pregnancy. When specific cut-off points were investigated, these associations were not statistically significant.

**Supplementary Information:**

The online version contains supplementary material available at 10.1007/s00404-025-08299-7.

## What does this study adds to the clinical work

**Table:** 

Our study demonstrated that, in twin pregnancies, low first-trimester PAPP-A levels were related with preterm birth, whereas high levels were associated with hypertensive disorders of pregnancy. Larger studies can clarify whether these findings will find their way to be used in the first-trimester predictive models.	

## Introduction

Multiple pregnancy carries increased risk of obstetric complications, including preterm birth (PTB), fetal growth restriction (FGR), and hypertensive disorders of pregnancy (HDP), that may result in substantial perinatal morbidity and mortality [1, 2]. In singleton pregnancies, the maternal serum biomarkers used in the first-trimester screening for aneuploidies, particularly PAPP-A (pregnancy-associated plasma protein-A), may be used as predictors of adverse perinatal outcomes such as pre-eclampsia (PET), fetal growth restriction (FGR), and preterm birth (PTB) [3–8]. In twin pregnancies however, there is a scarcity of data regarding the association of first-trimester biomarkers with unfavorable pregnancy outcomes as previous studies have yielded conflicting results [7, 8]. Based on current research concerning twin pregnancies, low PAPP-A levels seem to correlate with preterm birth prior to 32 and 34 weeks of gestation, as well as FGR or small for gestational age (SGA) fetus/neonate [8].

The main objective of this study was to assess the possible correlation of low PAPP-A levels with the subsequent development of preterm premature rupture of membranes (PPROM), preterm birth (PTB) at < 32, < 34, and < 36 weeks of gestation, gestational diabetes (GDM), hypertensive disorders of pregnancy (HDP) such as early and late-onset pre-eclampsia or gestational hypertension, intrauterine death (IUD) of at least one twin (s-IUD), birthweight discordance (BWD) of more than 25% among the fetuses and composite adverse pregnancy outcome (which included PPROM, PTB < 36 weeks, HDP, and GDM).

## Materials and methods

This is a retrospective cohort analysis of twin pregnancies that were examined and followed up in four fetal medicine centers over a period of 11 years, spanning from September 2014 to March 2025. The study was granted approval by the local Ethics Committee (97/2015). The recruitment involved women aged > 18 years old with twin pregnancies presenting for the routine first-trimester examination at 11^+0^–13^+6^ weeks. Only pregnancies with two viable fetuses were included. Cases with monoamniotic twins, chromosomal and/or severe fetal structural abnormalities, single fetal demise prior to 24 weeks, twin-to-twin transfusion syndrome (TTTS), and twin anemia-polycythemia sequence (TAPS) were excluded.

Maternal demographic characteristics as well as medical and obstetric history were recorded in the first visit (Astraia software, Nexus/Astraia GmbH).

Gestational age was determined based on the last menstrual cycle and was validated or adjusted by measuring the fetal crown–rump length of the larger twin during the first-trimester ultrasound. In cases of assisted reproductive technology (ART), the dating was based on the date of embryo transfer or the estimated due date (EDD) provided by the ART center.

Chorionicity was determined by the presence of lambda or T-sign for dichorionic (DC) and monochorionic (MC) twins, respectively [9].

The examination composed of the ultrasound examination of the fetal anatomy [10] and the markers of aneuploidy and the measurement of the maternal serum concentration of PAPP-A and free b-hCG by one of two automated biochemical analyzers: Kryptor (Thermo Fisher Scientific, Clinical Diagnostics, Brahms GmbH, Hennigsdorf, Germany) or Cobas (Roche Diagnostics, Basel, Switzerland) [9]. The concentrations of the first-trimester biochemical analytes were converted to multiples of the median (MoM) utilizing the FMF software (https://www.fetalmedicine.org/).

HDP were defined in accordance with the International Society for the Study of Hypertension in Pregnancy (ISSHP) [11]. According to the national guidelines, all women had glucose tolerance test at 24–28 weeks of pregnancy. The diagnosis of GDM was established in women who had at least one abnormal result after a 75 g 2-h OGTT: fasting ≥ 92 mg/dL, 1-h ≥ 180 mg/dL, 2-h ≥ 153 mg/dL [12]. Preterm birth was defined as any birth occurring up to 31 weeks and 6 days (PTB < 32), any birth occurring before 33 weeks and 6 days (PTB < 34) or any birth occurring before 35 weeks and 6 days (PTB < 36). Early-onset and late-onset pre-eclampsia was defined as disease occurring before (< 34 weeks) or after (≥ 34 weeks) 34 gestational weeks. PPROM was defined as rupture of the membranes that occurred before 36 weeks and was confirmed by physical examination.

The evaluated outcomes included PPROM, PTB earlier than 32, 34, and 36 weeks, iatrogenic PTB < 36 weeks, GDM, any PET, early- and late-onset PET (< 34 and) or GH, combined HDP (PET and GH), selective-IUD or IUD of both, BWD of more than 25% among the fetuses and composite adverse pregnancy outcome (which included PPROM, PTB < 36 weeks, HDP, and GDM).

The pregnancy outcomes were ascertained after communication with the physician or the mother.

### Statistical analysis

Quantitative variables were expressed as mean values (Standard Deviation) and as median (interquartile range), while categorical variables were expressed as absolute and relative frequencies. We investigated the association of PAPP-A MoM with PPROM, PTB, PET, early-onset PET, late-onset PET, Gestational Hypertension, GDM, BW discordance ≥ 25%, s-IUD, and IUD of both fetuses via logistic regression analysis, from which odds ratios (OR) with 95% confidence intervals (95% CI) emerged. Maternal age, chorionicity and conception were also taken into account in the logistic analyses. In cases with zero events, Firth penalized logistic regression was applied, which overcomes the limitations of traditional logistic regression when working with small datasets, particularly those characterized by rare events and complete separation [13, 14]. Statistical significance was set at *p* < 0.05, and analyses were conducted using SPSS statistical software (version 27.0).

## Results

Data from 454 Caucasian women were analyzed. The demographic characteristics and the pregnancy outcomes are presented in Tables 1 and 2, respectively.

**Table 1:** Demographic characteristics of the study population (*N* = 454 twin pregnancies)

			*n*	%
Maternal characteristics	Conception	Spontaneous	189	41.6
		Assisted	265	58.4
	Chorionicity	Dichorionic	392	86.3
		Monochorionic	62	13.7
	Nulliparous		331	72.9
	Smoking		43	9.5
	Pre-existing diabetes		15	3.3
	Pre-existing hypertension		4	0.9
	Systemic lupus erythematosus	4	0.9	
	Antiphospholipid syndrome	2	0.4	
	History of pre-eclampsia	3	0.7	
	Previous low birth weight	0	0.0	
			Mean (SD)	Median (IQR)
	Age (years)		35.7 (4.3)	35.5 (33–38.7)
	Weight (kg)		69.8 (15.2)	67 (59.1–77)
	Maternal height (cm)		166.1 (5.9)	166 (162–170)
	BMI (1st trimester)		25.3 (5.1)	24.0 (21.6–27.9)
Biochemical parameters	PAPP-A MoM		1.35 (0.65)	1.23 (0.89–1.71)
	Free β-hCG MoM		1.22 (0.77)	1.05 (0.73–1.53)
Biophysical parameters	MAP		88.6 (8.2)	88.46 (83.33–93.67)
	Mean Uterine Artery PI		1.5 (0.45)	1.43 (1.19–1.74)

**Table 2:** Obstetric outcomes of the study population (*N* = 454 twin pregnancies)

Outcomes	*n*	%
PPROM	54	11.9
PTB < 36 weeks	214	47.1
Iatrogenic PTB < 36 weeks^1^	77	62.1
PTB < 34 weeks	102	22.5
PTB < 32 weeks	57	12.6
PET	20	4.4
Early onset PET < 34 weeks	9	2.0
Late onset PET > 34 weeks	11	2.4
Gestational Hypertension	12	2.6
GDM	61	13.4
BW discordance ≥ 25%	10	2.2
s-IUD	6	1.3
IUD both	4	0.9
HDP	31	6.8
Composite Adverse pregnancy outcomes	263	57.9

^1^information available in 124 cases that delivered before 36 weeks

*PTB* Preterm Birth, *PET* Pre-eclampsia, *PPROM* Preterm premature rupture of membranes, *GDM* Gestational diabetes, *IUD* Intrauterine death, *sIUD* Intrauterine death of at least one twin, *BW* Birthweight, *HDP* Hypertensive disorders of the pregnancy includes PET and Gestational Hypertension; Composite Adverse Pregnancy outcome includes PPROM, PTB < 36 weeks, PET, Gestational Hypertension, and GDM

Logistic regression analysis using PAPP-A MoM values as a continuous variable showed a significant association of PAPP-A with PTB < 32 weeks (OR = 0.60; *p* = 0.048), PET (OR = 1.79; *p* = 0.030), and hypertensive disorders of pregnancy (OR = 1.66; *p* = 0.031) (Table 3). The aforementioned results remained significant after adjusting for age and chorionicity.

**Table 3:** Logistic regression analysis presenting the association of PAPP-A MoM as a continuous variable with adverse pregnancy outcomes

		PAPP-A MoM		OR (95% CI) +	*P*	OR (95% CI) + +	*P*
		Mean (SD)	Median (IQR)				
PPROM	No	1.35 (0.66)	1.24 (0.91–1.70)	0.93 (0.60–1.46)	0.754	0.98 (0.62–1.54)	0.926
	Yes	1.32 (0.62)	1.22 (0.85–1.71)				
PTB < 36 weeks	No	1.39 (0.70)	1.24 (0.92–1.74)	0.83 (0.62–1.11)	0.206	0.78 (0.58–1.05)	0.107
	Yes	1.31 (0.60)	1.23 (0.87–1.65)				
PTB < 34 weeks	No	1.36 (0.66)	1.25 (0.91–1.73)	0.90 (0.63–1.27)	0.547	0.81 (0.57–1.17)	0.266
	Yes	1.31 (0.63)	1.13 (0.87–1.63)				
PTB < 32 weeks	No	1.37 (0.66)	1.26 (0.92–1.73)	0.60 (0.37–0.99)	0.048	0.56 (0.34–0.94)	0.028
	Yes	1.19 (0.56)	1.05 (0.82–1.51)				
PET	No	1.33 (0.66)	1.22 (0.89–1.68)	1.79 (1.06–3.04)	0.030	1.65 (0.96–2.83)	0.071
	Yes	1.67 (0.49)	1.69 (1.31–2.04)				
Early onset PET < 34 weeks	No	1.34 (0.66)	1.23 (0.89–1.69)	1.72 (0.83–3.57)	0.147	1.53 (0.69–3.40)	0.295
	Yes	1.66 (0.40)	1.64 (1.54–2.04)				
Late onset PET > 34 weeks	No	1.34 (0.65)	1.23 (0.89–1.68)	1.75 (0.90–3.41)	0.099	1.63 (0.84–3.19)	0.151
	Yes	1.67 (0.57)	1.88 (1.05–2.13)				
Gestational Hypertension	No	1.34 (0.65)	1.23 (0.89–1.69)	1.30 (0.60–2.79)	0.503	1.28 (0.60–2.73)	0.532
	Yes	1.47 (0.59)	1.32 (0.98–1.90)				
GDM	No	1.35 (0.66)	1.24 (0.89–1.71)	0.97 (0.64–1.48)	0.898	0.92 (0.60–1.40)	0.682
	Yes	1.34 (0.63)	1.23 (0.94–1.61)				
BW discordance ≥ 25%	No	1.35 (0.65)	1.24 (0.90–1.71)	0.84 (0.30–2.37)	0.748	0.76 (0.27–2.17)	0.611
	Yes	1.28 (0.59)	1.09 (0.82–1.64)				
s-IUD	No	1.35 (0.65)	1.23 (0.90–1.70)	0.79 (0.20–3.08)	0.736	0.75 (0.19–2.96)	0.677
	Yes	1.26 (0.67)	1.16 (0.64–1.79)				
IUD both	No	1.35 (0.66)	1.24 (0.89–1.71)	0.64 (0.11–3.77)	0.624	0.72 (0.11–4.91)	0.735
	Yes	1.19 (0.25)	1.08 (1.05–1.34)				
Ηypertensive disorders of pregnancy	No	1.33 (0.66)	1.22 (0.89–1.66)	1.66 (1.05–2.63)	0.031	1.56 (1.03–2.50)	0.041
	Yes	1.60 (0.53)	1.67 (1.05–2.04)				
Composite Adverse pregnancy outcomes	No	1.38 (0.71)	1.23 (0.92–1.74)	0.89 (0.67–1.18)	0.423	0.83 (0.62–1.11)	0.201
	Yes	1.33 (0.61)	1.23 (0.89–1.66)				
Iatrogenic PTB < 36 weeks	No	1.32 (0.64)	1.14 (0.79–1.71)	0.94 (0.52–1.69)	0.825	0.63 (0.32–1.25)	0.185
	Yes	1.30 (0.59)	1.24 (0.82–1.64)				

+ Crude Odds Ratio for 1 unit increase of PAPP-A MoM (95% Confidence Interval)

+ + Odds Ratio for 1 unit increase of PAPP-A MoM adjusted for age, chorionicity, and conception (95% Confidence Interval)

*PTB* Preterm birth, *PET* Pre-eclampsia, *PPROM* Preterm premature rupture of membranes, *GDM* Gestational diabetes, *IUD* Intrauterine death, *sIUD* Intrauterine death of at least one twin, *BW* Birthweight, *HDP* Hypertensive disorders of the pregnancy includes PET and Gestational Hypertension; Composite Adverse Pregnancy outcome includes PPROM, PTB < 36 weeks, PET, Gestational Hypertension, and GDM

After adjusting for age, chorionicity and conception, a significant association of PAPP-A with PTB < 32 weeks (OR = 0.56; *p* = 0.028) and hypertensive disorders of pregnancy (OR = 1.56; *p* = 0.041) remained.

Neither low PAPP-A MoM < 5th percentile and MoM < 10th percentile nor high PAPP-A > 90th and > 95th centile were significantly associated with any of the investigated outcomes (Supplementary Tables S1–S4). The findings remained non-significant after adjustment for maternal age and chorionicity.

## Discussion

### Principal findings of our study

We observed a significant association of PAPP-A MoM with preterm birth < 32 weeks and hypertensive disorders of pregnancy (Table 3). Higher PAPP-A was associated with lower risk of PTB < 32 weeks and higher risk of hypertensive disorders of pregnancy.

However, when specific cut-off points were used, such as PAPP-A < 5th, < 10th, > 90th or < 95th centile, this association was lost. Similarly, there was no significant association with the other adverse pregnancy outcomes.

### Results in the context of what is known

Several studies have investigated the association of low PAPP-A levels with adverse perinatal outcomes in twin pregnancies with our recent review summarizing the available evidence [2].

The majority of the studies shows no association with PTB, PET, and GH [15, 16, 18]. However, all those studies used PAPP-A as a dichotomous variable, examining cut-off points such as the 5th and 10th centile of the measurements, and it is possible that an analysis using PAPP-A as a continuous variable would produce different results. Two studies [15, 16] demonstrated non-significant correlation of low PAPP-A levels with PTB and GH, although regarding PTB Laughon et al. observed a trend, with deliveries occurring before 32 weeks being nearly three times more prevalent in women with PAPP-A concentrations below the 25th percentile. Similarly, a Danish group demonstrated non-significant results on the correlation of low PAPP-A levels and BWD when assessing data derived from 762 MCDA pregnancies [17].

Contrary to the above, Saletra- Bielinska et al. [18] associated both low (< 10th centile) and high levels (> 90th centile) of first-trimester PAPP-A with the subsequent development of PTB with their results being corroborated by the findings of Rosner et al. [19]. Rosner et al. observed a greater incidence of preterm delivery in twin pregnancies with low PAPP-A, defined as < 0.42 MoM. The analysis of their cohort of 340 patients revealed an elevated risk of preterm delivery (RR 5.56; 95% CI 1.5–20.1) in women exhibiting low first-trimester PAPP-A concentrations. In addition, a recent study by Queirós et al. showed a significant association of low PAPP-A and iatrogenic extreme prematurity (PTB < 32 weeks) in cases when PTB was combined with FGR and/or GH [1]. In accordance, our data demonstrated a correlation of lower PAPP-A values with PTB < 32 weeks, although we did not observe this to be associated with iatrogenic deliveries.

### Clinical and research implications

The association of lower levels of PAPP-A with higher risk of PTB < 32 may be explained by its inherent downregulating activity on IGF2 bioavailability, subsequently leading to compromised placentation and placental ischemia, irregularities that have been associated with preterm birth [20–22].

The correlation of higher PAPPA levels with hypertensive disorders of pregnancy and pre-eclampsia has not been reported previously as indeed PAPP-A levels have not been reported to be predictive of pre-eclampsia in previous studies. Actually, as this correlation was only observed when PAPP-A was considered as a continuous variable, it is possible that it has not been spotted because only low PAPP-A levels were investigated. This association seems puzzling because in singleton pregnancies it is low PAPP-A levels that predict PET. However, even in singletons, low PAPP-A is mainly associated with early onset PET commonly accompanied by FGR [23, 24] and much less with late-onset PET and we know that the underlying mechanisms for the two conditions are probably different, although overlapping certainly occurs [25, 26]. Late onset PET is characterized by enhanced maternal reaction to the pregnancy as expressed by enhanced cardiac output and normal or enhanced fetal growth with no pathologic signs of suboptimal placentation but rather large placentas [27, 28]. As the increased risk of PET in twins is probably related mostly to the increased placental mass and less so to the faulty placentation, we may explain our finding as a sign of ‘too much placenta’ leading to PET.

PAPP-A seems to be an essential component of pregnancy since it regulates placental function and development by controlling insulin-like growth factor activity and, as such, placental volume and possibly the trophoblastic tissue itself [29]. In singleton pregnancies, PAPP-A is an important predictor for PET and fetal growth restriction when combined with other demographic, biophysical and biochemical indices [30]. Unfortunately, those models underperform in twins probably because the presence of two feto-placental units mitigates the contribution of the biochemical markers but also because the underlying mechanisms have different impact on the development of the disease compared to singleton pregnancies.

Based on our study’s findings, it seems necessary to discuss the potential complications of both low and high PAPP-A levels with parents, emphasizing on the importance of a strict follow-up of the index pregnancy and highlighting potential worrisome signs and symptoms of preterm labor and hypertensive disorders of pregnancy. The consultation should address the importance of the second trimester cervical assessment, as this remains the best factor to predict preterm birth, independent of other risk factors [31, 32]. Furthermore, couples should be informed of the increased incidence of pre-eclampsia in twin pregnancies [33], as well as, alarming signs (high blood pressure) and symptoms (headache, vision changes and upper abdominal pain) that the condition is associated with. However, given the fact that multiple pregnancies are susceptible to increased rates of adverse pregnancy outcomes, future studies need to focus on investigating and formulating predictive models to guide management and clinical decisions.

### Strengths and limitations

The present study examined a large sample size of twin pregnancies, and the fetal ultrasound examinations were conducted by experienced fetal medicine experts which strengthens the accuracy of our data. The use of PAPP-A as a continuous variable revealed an association with hypertensive disorders of pregnancy that has not reported previously.

However, our cohort was retrospective and we could not distinguish between spontaneous and iatrogenic preterm deliveries in all cases. The sample size of MCDA pregnancies was small and a sub-analysis based on chorionicity was not meaningful. Furthermore, the majority of our population received aspirin from as early as the first trimester, and this may potentially have altered the true incidence of some outcomes.

## Conclusions

Our study demonstrated that low first-trimester PAPP-A levels were related with preterm birth, whereas high levels were associated with hypertensive disorders of pregnancy. Larger studies can clarify whether these findings will find their way to be used in the first-trimester predictive models.

## Supplementary Information

Below is the link to the electronic supplementary material.

Supplementary file1 (DOCX 33 KB)

## Data Availability

Data is provided within the manuscript or supplementary information files.
