# Supplementary material for: Low PAPP-A levels and their association with adverse perinatal outcomes in twin pregnancies
Source: Arch Gynecol Obstet. 2026 Jan 19;313(1):51. doi: 10.1007/s00404-025-08299-7 (PMC12816117; doi:10.1007/s00404-025-08299-7)
Supplement: Supplementary file 1 — Supplementary file1 (DOCX 33 KB) [file 404_2025_8299_MOESM1_ESM.docx]

**Table S1.** Logistic regression analysis presenting the association of low PAPP-A MoM<5th percentile with adverse pregnancy outcomes.

|  | **PAPP-A MoM** | |  | | | |
| --- | --- | --- | --- | --- | --- | --- |
| **Outcome** | **>5th percentile** | **<5th percentile** | **OR (95% CI)+** | **P** | **OR (95% CI)++** | **P** |
| PPROM | 52 (12.1) | 2 (8.3) | 0.66 (0.15 ─ 0.29) | 0.582 | 0.64 (0.15 ─ 2.84) | 0.561 |
| PTB<36weeks | 202 (47.0) | 12 (50.0) | 1.13 (0.50 ─ 2.57) | 0.773 | 1.09 (0.48 ─ 2.49) | 0.836 |
| PTB<34weeks | 97 (22.6) | 5 (20.8) | 0.90 (0.33 ─ 2.48) | 0.844 | 0.90 (0.33 ─ 2.48) | 0.844 |
| PTB<32weeks | 53 (12.3) | 4 (16.7) | 1.42 (0.47 ─ 4.32) | 0.534 | 0.84 (0.31 ─ 2.34) | 0.743 |
| PET | 20 (4.7) | 0 (0.0) | 0.41 (0.02 ─ 6.96) | 0.536 | 0.39 (0.02 ─ 6.67) | 0.516 |
| Early onset PET<34weeks | 9 (2.1) | 0 (0.0) | 0.91 (0.05 ─ 16.02) | 0.946 | 0.75 (0.04 ─ 13.43) | 0.843 |
| Late onset PET>34 weeks | 11 (2.6) | 0 (0.0) | 0.74 (0.04 ─ 13.01) | 0.840 | 0.77 (0.04 ─ 13.53) | 0.859 |
| Gestational Hypertension | 12 (2.8) | 0 (0.0) | 0.68 (0.04 ─ 11.88) | 0.794 | 0.71 (0.04 ─ 12.36) | 0.813 |
| GDM | 58 (13.5) | 3 (12.5) | 0.92 (0.27 ─ 3.17) | 0.890 | 1.42 (0.46 ─ 4.34) | 0.539 |
| BW discordance ≥ 25% | 10 (2.3) | 0 (0.0) | 0.82 (0.05 ─ 14.36) | 0.890 | 0.74 (0.04 ─ 13.27) | 0.840 |
| s-IUD | 6 (1.4) | 0 (0.0) | 1.33 (0.07 ─ 24.35) | 0.846 | 1.16 (0.06 ─ 21.5) | 0.921 |
| IUD both | 4 (0.9) | 0 (0.0) | 1.93 (0.10 ─ 36.95) | 0.661 | 1.06 (0.05 ─ 23.79) | 0.972 |
| ΗDP | 31 (7.2) | 0 (0.0) | 0.26 (0.02 – 4.36) | 0.348 | 0.27 (0.02 – 4.34) | 0.346 |
| Composite Adverse pregnancy outcomes | 247 (57.4) | 16 (66.7) | 1.48 (0.62 – 3.54) | 0.376 | 1.41 (0.59 – 3.37) | 0.446 |
| Iatrogenic PTB<36 weeks | 73 (61.9) | 4 (66.7) | 1.23 (0.22 – 7.01) | 0.813 | 1.35 (0.23 – 7.92) | 0.739 |

+Crude Odds Ratio for having PAPP-A MoM<5th percentile (95% Confidence Interval)

++ Odds Ratio for having PAPP-A MoM<5th percentile adjusted for age and chorionicity (95% Confidence Interval)

Abbreviations. PTB= Preterm Birth, PET=Preeclampsia , PPROM= preterm premature rupture of membranes , GDM= gestational diabetes ,IUD= intrauterine death, sIUD= intrauterine death of at least one twin, BW= birthweight , HDP= hypertensive disorders of the pregnancy includes PET and Gestational Hypertension; Composite Adverse Pregnancy outcome includes PPROM, PTB<36weeks, PET, Gestational Hypertension and GDM

**Table S2.** Logistic regression analysis presenting the association of low PAPP-A MoM<10th percentile with adverse pregnancy outcomes.

|  | **PAPP-A MoM** | |  | | | |
| --- | --- | --- | --- | --- | --- | --- |
| **Outcome** | **>10th percentile** | **<10th percentile** | **OR (95% CI)+** | **P** | **OR (95% CI)++** | **P** |
| PPROM | 48 (11.8) | 6 (13.0) | 1.12 (0.45 ─ 2.79) | 0.800 | 1.14 (0.46 ─ 2.86) | 0.773 |
| PTB<36weeks | 191 (46.8) | 23 (50.0) | 1.14 (0.62 ─ 2.09) | 0.682 | 1.13 (0.62 ─ 2.09) | 0.685 |
| PTB<34weeks | 94 (23.0) | 8 (17.4) | 0.70 (0.32 ─ 1.56) | 0.386 | 0.70 (0.31 ─ 1.55) | 0.375 |
| PTB<32weeks | 52 (12.7) | 5 (10.9) | 0.83 (0.32 ─ 2.21) | 0.716 | 0.83 (0.31 ─ 2.20) | 0.706 |
| PET | 20 (4.9) | 0 (0.0) | 0.20 (0.01 ─ 3.43) | 0.269 | 0.21 (0.01 ─ 3.48) | 0.274 |
| Early onset PET<34weeks | 9 (2.2) | 0 (0.0) | 0.45 (0.03 ─ 7.9) | 0.587 | 0.44 (0.03 ─ 7.75) | 0.577 |
| Late onset PET>34 weeks | 11 (2.7) | 0 (0.0) | 0.37 (0.02 ─ 6.41) | 0.496 | 0.38 (0.02 ─ 6.56) | 0.506 |
| Gestational Hypertension | 12 (2.9) | 0 (0.0) | 0.34 (0.02 ─ 5.86) | 0.458 | 0.35 (0.02 ─ 5.99) | 0.468 |
| GDM | 56 (13.7) | 5 (10.9) | 0.77 (0.29 ─ 2.02) | 0.591 | 0.78 (0.29 ─ 2.06) | 0.612 |
| BW discordance ≥ 25% | 9 (2.2) | 1 (2.2) | 0.99 (0.12 ─ 7.96) | 0.989 | 1.00 (0.12 ─ 8.18) | 0.998 |
| s-IUD | 5 (1.2) | 1 (2.2) | 1.79 (0.2 ─ 15.67) | 0.598 | 1.77 (0.20 ─ 15.57) | 0.605 |
| IUD both | 4 (1.0) | 0 (0.0) | 0.97 (0.05 ─ 18.24) | 0.982 | 0.78 (0.04 ─ 15.67) | 0.873 |
| Ηypertensive disorders of pregnancy | 31 (7.6) | 0 (0.0) | 0.13 (0.01 – 2.14) | 0.153 | 0.13 (0.01 – 2.18) | 0.156 |
| Composite Adverse pregnancy outcomes | 235 (57.6) | 28 (60.9) | 1.15 (0.61 – 2.14) | 0.670 | 1.15 (0.62 – 2.16) | 0.655 |
| Iatrogenic PTB<36 weeks | 69 (62.2) | 8 (61.5) | 0.97 (0.30 – 3.17) | 0.965 | 1.00 (0.30 – 3.32) | 0.996 |

+Crude Odds Ratio for having PAPP-A MoM<10h percentile (95% Confidence Interval)

++ Odds Ratio for having PAPP-A MoM<10th percentile adjusted for age and chorionicity (95% Confidence Interval)

Abbreviations. PTB= Preterm Birth, PET=Preeclampsia , PPROM= preterm premature rupture of membranes , GDM= gestational diabetes ,IUD= intrauterine death, sIUD= intrauterine death of at least one twin, BW= birthweight , HDP= hypertensive disorders of the pregnancy includes PET and Gestational Hypertension; Composite Adverse Pregnancy outcome includes PPROM, PTB<36weeks, PET, Gestational Hypertension and GDM

**Table S3.** Logistic regression analysis presenting the association of PAPP-A MoM ≥90th percentile with study outcomes.

|  | **PAPP-A MoM** | |  |  |  |  |
| --- | --- | --- | --- | --- | --- | --- |
|  | **<90th percentile** | **>=90th percentile** |  |  |  |  |
|  | **n (%)** | **n (%)** | **OR (95% CI)+** | **P** | **OR (95% CI)++** | **P** |
| PPROM | 48 (11.7) | 6 (13.3) | 1.16 (0.47 ─ 2.88) | 0.754 | 1.11 (0.45 ─ 2.79) | 0.816 |
| PTB<36weeks | 196 (47.9) | 18 (40.0) | 0.72 (0.39 ─ 1.36) | 0.314 | 0.72 (0.39 ─ 1.36) | 0.312 |
| PTB<34weeks | 90 (22) | 12 (26.7) | 1.29 (0.64 ─ 2.60) | 0.478 | 1.30 (0.64 ─ 2.62) | 0.472 |
| PTB<32weeks | 52 (12.7) | 5 (11.1) | 0.86 (0.32 ─ 2.27) | 0.758 | 0.87 (0.33 ─ 2.30) | 0.775 |
| PET | 18 (4.4) | 2 (4.4) | 1.01 (0.23 ─ 4.50) | 0.989 | 0.97 (0.22 ─ 4.32) | 0.963 |
| Early onset PET<34weeks | 9 (2.2) | 0 (0.0) | 0.46 (0.03 - 8.09) | 0.598 | 0.43 (0.02 - 7.60) | 0.568 |
| Late onset PET>34 weeks | 9 (2.2) | 2 (4.4) | 2.07 (0.43 ─ 9.88) | 0.363 | 2.02 (0.42 ─ 9.71) | 0.381 |
| Gestational Hypertension | 10 (2.4) | 2 (4.4) | 1.86 (0.39 ─ 8.75) | 0.434 | 1.81 (0.38 ─ 8.59) | 0.455 |
| GDM | 55 (13.4) | 6 (13.3) | 0.99 (0.40 ─ 2.45) | 0.983 | 0.94 (0.38 ─ 2.35) | 0.903 |
| BW discordance ≥ 25% | 9 (2.2) | 1 (2.2) | 1.01 (0.13 ─ 8.16) | 0.992 | 0.93 (0.11 ─ 7.56) | 0.943 |
| s-IUD | 5 (1.2) | 1 (2.2) | 1.84 (0.21 ─ 16.07) | 0.583 | 1.82 (0.21 ─ 16.05) | 0.589 |
| IUD both | 4 (1) | 0 (0.0) | 0.99 (0.05 - 18.69) | 0.995 | 1.23 (0.06 - 24.31) | 0.890 |
| Ηypertensive disorders of pregnancy | 27 (6.6) | 4 (8.9) | 1.38 (0.46 ─ 4.14) | 0.565 | 1.33 (0.44 ─ 4.02) | 0.610 |
| Composite Adverse pregnancy outcomes | 237 (57.9) | 26 (57.8) | 0.99 (0.53 ─ 1.85) | 0.983 | 0.98 (0.52 ─ 1.83) | 0.946 |
| Iatrogenic onset | 72 (63.7) | 5 (45.5) | 0.47 (0.14 ─ 1.65) | 0.241 | 0.48 (0.14 ─ 1.72) | 0.262 |

+Crude Odds Ratio for having PAPP-A MoM ≥10^h^ percentile (95% Confidence Interval);

++ Odds Ratio for having PAPP-A MoM ≥10^th^ percentile adjusted for age and chorionicity (95% Confidence Interval)

Abbreviations. PTB= Preterm Birth, PET=Preeclampsia , PPROM= preterm premature rupture of membranes , GDM= gestational diabetes ,IUD= intrauterine death, sIUD= intrauterine death of at least one twin, BW= birthweight , HDP= hypertensive disorders of the pregnancy includes PET and Gestational Hypertension; Composite Adverse Pregnancy outcome includes PPROM, PTB<36weeks, PET, Gestational Hypertension and GDM

**Table S4.** Logistic regression analysis presenting the association of PAPP-A MoM ≥95th percentile with study outcomes.

|  | **PAPP-A MoM** | |  |  |  |  |
| --- | --- | --- | --- | --- | --- | --- |
|  | **<95th percentile** | **>=95th percentile** |  |  |  |  |
|  | **n (%)** | **n (%)** | **OR (95% CI)+** | **P** | **OR (95% CI)++** | **P** |
| PPROM | 51 (11.9) | 3 (12.0) | 1.01 (0.29 ─ 3.50) | 0.987 | 0.98 (0.28 ─ 3.42) | 0.969 |
| PTB<36weeks | 206 (48.0) | 8 (32.0) | 0.51 (0.22 ─ 1.21) | 0.125 | 0.49 (0.21 ─ 1.17) | 0.109 |
| PTB<34weeks | 96 (22.4) | 6 (24.0) | 1.10 (0.43 ─ 2.82) | 0.850 | 1.04 (0.40 ─ 2.70) | 0.932 |
| PTB<32weeks | 55 (12.8) | 2 (8.0) | 0.59 (0.14 ─ 2.58) | 0.484 | 0.59 (0.13 ─ 2.57) | 0.479 |
| PET | 19 (4.4) | 1 (4.0) | 0.90 (0.12 ─ 7.00) | 0.919 | 0.85 (0.11 ─ 6.69) | 0.875 |
| Early onset PET<34weeks | 9 (2.1) | 0 (0.0) | 0.87 (0.05 - 15.33) | 0.923 | 0.70 (0.04 - 12.71) | 0.808 |
| Late onset PET>34 weeks | 10 (2.3) | 1 (4.0) | 1.75 (0.21 ─ 14.21) | 0.602 | 1.84 (0.22 ─ 15.20) | 0.573 |
| Gestational Hypertension | 12 (2.8) | 0 (0.0) | 0.65 (0.04 - 11.38) | 0.771 | 0.68 (0.04 - 11.91) | 0.792 |
| GDM | 58 (13.5) | 3 (12.0) | 0.87 (0.25 ─ 3.01) | 0.829 | 0.83 (0.24 ─ 2.89) | 0.764 |
| BW discordance ≥ 25% | 10 (2.3) | 0 (0.0) | 0.78 (0.04 - 13.75) | 0.867 | 0.66 (0.04 - 11.94) | 0.781 |
| s-IUD | 6 (1.4) | 0 (0.0) | 1.28 (0.70 - 23.31) | 0.869 | 1.19 (0.06 - 21.86) | 0.909 |
| IUD both | 4 (0.9) | 0 (0.0) | 1.85 (0.10 - 35.39) | 0.682 | 1.50 (0.07 - 34.12) | 0.799 |
| Ηypertensive disorders of pregnancy^1^ | 30 (7.0) | 1 (4.0) | 0.55 (0.07 ─ 4.24) | 0.570 | 0.54 (0.07 ─ 4.21) | 0.560 |
| Composite Adverse pregnancy outcomes^2^ | 251 (58.5) | 12 (48.0) | 0.65 (0.29 ─ 1.47) | 0.304 | 0.62 (0.27 ─ 1.41) | 0.254 |
| Iatrogenic onset^3^ | 75 (63.0) | 2 (40.0) | 0.39 (0.06 ─ 2.43) | 0.314 | 0.42 (0.06 ─ 2.82) | 0.375 |

+Crude Odds Ratio for having PAPP-A MoM ≥10^h^ percentile (95% Confidence Interval);

++ Odds Ratio for having PAPP-A MoM ≥10^th^ percentile adjusted for age and chorionicity (95% Confidence Interval)

Abbreviations. PTB= Preterm Birth, PET=Preeclampsia , PPROM= preterm premature rupture of membranes , GDM= gestational diabetes ,IUD= intrauterine death, sIUD= intrauterine death of at least one twin, BW= birthweight , HDP= hypertensive disorders of the pregnancy includes PET and Gestational Hypertension; Composite Adverse Pregnancy outcome includes PPROM, PTB<36weeks, PET, Gestational Hypertension and GDM
